# Exergames improves cognitive functions in adolescents with depression: study protocol of a prospective, assessor-blind, randomized controlled trial

**DOI:** 10.1186/s12888-023-04967-7

**Published:** 2023-07-13

**Authors:** De-Quan Wang, Jing-Jing Zhang, Jian-Ning Chen, Ren-Yu Li, Yi-Xiang Luo, Wei Deng

**Affiliations:** 1 Mental Health Center, West China Hospital, Sichuan University, Chengdu, 610041 China; 2 School of Clinical Medicine, Sichuan University, Chengdu, 610041 China; 3 Affiliated Mental Health Center & Hangzhou Seventh People’s Hospital and School of Brain Science and Brain Medicine, Zhejiang University School of Medicine, Hangzhou, 310058 China; 4 Alibaba Group, Hangzhou, 311121 China; 5 Liangzhu Laboratory, MOE Frontier Science Center for Brain Science and Brain-machine Integration, State Key Laboratory of Brain-machine Intelligence, Zhejiang University, 1369 West Wenyi Road, Hangzhou, 311121 China

**Keywords:** Exergames, Depression, Adolescents, Cognitive functions, Exercise, Randomized clinical trial, Clinical trial protocol

## Abstract

**Background:**

Depression is a condition that imposes a significant disease burden, with cognitive impairment being one of its costly symptoms. While cognitive rehabilitation is crucial, it is also challenging. Although some studies have investigated the impact of exergames on cognitive function improvement, these have primarily focused on the elderly population, with limited attention given to individuals with depression. Consequently, this study aims to investigate the effects of exergames on cognitive functions in adolescents with depression and compare the effectiveness of exergames with traditional exercise.

**Method:**

The present investigation is a single-center randomized controlled trial that employs the ANOVA method to calculate the sample size using G*Power software, assuming a 25% dropout rate. The study enrolls fifty-four eligible patients with depression who are randomly allocated to one of three treatment groups: the exergames group, which receives standard treatment and exergames intervention; the exercise group, which receives standard treatment and traditional exercise intervention; and the control group, which receives standard treatment exclusively. The study provides a comprehensive regimen of 22 supervised exercise and exergame sessions over an 8-week period, with a frequency of twice per week for the initial two weeks and three times per week for the subsequent six weeks. The researchers gather cognitive, mood, and sleep metrics at the onset of the first week, as well as at the conclusion of the fourth and eighth weeks. The researchers employ a wearable device to track participants' heart rate during each intervention session and evaluate the Borg Rating of Perceived Exertion scale at the conclusion of each session.

**Discussion:**

The findings from this study make several contributions to the current literature. First, this study comprehensively reports the efficacy of an exergames intervention for multidimensional symptoms in adolescents with depression. Second, this study also compares the efficacy of exergames with that of traditional exercise. These findings provide a theoretical basis for the use of exergames as an adjunctive intervention for depression and lay the groundwork for future research.

**Trial registration:**

This trial is registered with the Chinese Clinical Trials Registry (Registration number: ChiCTR2100052709; Registration Status: Prospective registration;) 3/11/2021, URL:   http://www.chictr.org.cn/edit.aspx?pid=135663&htm=4.

**Supplementary Information:**

The online version contains supplementary material available at 10.1186/s12888-023-04967-7.

## Introduction

Exergames represent a distinctive genre of physical activity that incorporates elements of video gaming. These games employ a human–machine interface that necessitates the simultaneous engagement of cognitive and motor skills [1]. Sensors such as accelerometers, strain gauges, and cameras are utilized to capture the movements of the gamer, which are subsequently transmitted wirelessly or via infrared to the device. These movements are then projected onto a screen, enabling the individual to engage with the virtual environment through bodily motions. Exergames require players to engage in physical movements and cognitive tasks, while receiving engaging and gratifying feedback, with the ultimate aim of enhancing physical fitness and cognitive abilities. As such, exergames serve not only as a source of amusement, but also as a training modality that confers benefits to both physical and cognitive well-being.

Depression is classified as an affective disorder. The World Health Organization has reported that depression affects over 300 million individuals globally, which is equivalent to roughly 5% of the world's population [2]. According to the projections of the World Health Organization, depression is anticipated to significantly contribute to the overall disease burden by the year 2030 [3]. Depressed patients frequently exhibit symptoms of low mood, diminished interest, and reduced cognitive processing speed. Furthermore, cognitive dysfunction is a prevalent feature among the majority of individuals with depression, primarily affecting executive function, memory, attention, and related domains [3–5]. The persistence of residual symptoms resulting in impaired cognitive functions can significantly impact patients' social interactions, family life, academic pursuits, and occupational endeavors. Cognitive rehabilitation is a crucial yet challenging aspect of treatment. Empirical evidence suggests that conventional depression therapies are inadequate in addressing the persistent cognitive impairments [6]. The effectiveness of cognitive behavioural therapy (CBT) in treating cognitive deficits is also limited [7]. The utilization of computer-based cognitive training has demonstrated the potential to enhance cognitive function [8], however, it is not exempt from certain constraints such as restricted availability, intricate operation, staffing prerequisites, and difficulties in ensuring patient compliance. Computer-based cognitive training is associated with high costs and necessitates patients to attend clinical settings, while the training process may become tedious, thereby posing a challenge for patients to maintain their engagement with the treatment. Furthermore, patients often require the accompaniment of family members to the hospital, which can serve as an additional impediment.

Nonetheless, an alternative approach to enhancing cognitive capacity is through physical exercise, a readily available and uncomplicated means. A plethora of evidence has substantiated the impact of exercise on brain plasticity, thereby yielding significant advantages for cognitive processes [8, 9]. Moreover, systematic reviews and meta-analyses conducted on adolescent populations have demonstrated that exercise serves as a viable intervention for mitigating depression and associated symptoms [10, 11]. According to a study published in The Lancet, involving a sample size of one million individuals, all types of exercise are found to be associated with a decrease in mental health burden, with a minimum reduction of 11.8% and a maximum reduction of 22.3%, compared to those who do not engage in exercise. This trend is also observed among individuals previously diagnosed with depression [12]. Despite the positive impact of exercise on mental health, participation rates remain low. A pooled analysis of 298 studies, encompassing 16 million adolescents from 146 countries, yielded the following results [13]. The research reveals that a significant proportion of teenagers globally, namely 81%, are not engaging in sufficient physical activity. Furthermore, 27 nations exhibit a higher prevalence of inadequate exercise, reaching 90%. Additionally, adolescents experiencing depression exhibit reduced adherence to exercise due to symptoms such as low mood, anhedonia, and decreased interest.

In recent years, there has been a notable surge in the prevalence and utilization of 'exergames' among adolescents. The study conducted by Rosenberg D. et al. offers an initial indication of the advantages of exergames in older adults with depression [14]. The present study has demonstrated a noteworthy amelioration in depressive symptoms and cognitive abilities subsequent to the implementation of exergames intervention. Additionally, a separate investigation has suggested that exergames have the potential to mitigate sedentary behavior in adolescents while enhancing their affective state and cognitive performance [15]. Exergames offer three primary benefits over conventional exercise in enhancing cognitive function. Firstly, exergames furnish greater cognitive stimulation by integrating cognitive demands into physical tasks [16]. Exergames are characterized by the presence of multisensory stimuli, encompassing auditory, visual, and somatosensory modalities. The reception and processing of this information necessitates the engagement of various cognitive functions, such as visual and verbal memory, attention, and other multidimensional cognitive processes. Secondly, exergames possess a higher degree of attraction and are capable of more efficiently engrossing individuals, thereby enhancing adherence. Empirical evidence further indicates that exergames facilitate superior adherence to exercise routines over conventional exercise in the extended period [17]. Thirdly, exergames exhibit a higher degree of interactivity in comparison to conventional sports, as they require participants to engage with the virtual environment through physical movements that stimulate cognitive faculties such as spatiotemporal perception, working memory, and executive function [16].

Consequently, the present study posits that exergames confer greater cognitive benefits to adolescents with depression compared to conventional exercise. Prior research on the cognitive impacts of exergames has predominantly focused on the elderly population. However, scant attention is given to the effects of exergames on the cognitive functions of adolescents experiencing depression. This study represents the first attempt to examine the distinct effects of exergames and conventional exercise on the cognitive functions, mood, and sleep quality of adolescents with depression. In order to obtain a comprehensive comprehension of the impacts, this research concentrates on three facets. Firstly, it assesses the cognitive advantages of a new intervention (exergames) in adolescents who suffer from depression. Secondly, it compares the effectiveness of exergames and exercise on cognition among the patients. Thirdly, it compares the clinical performance of exergames and exercise on mood and sleep enhancement in patients with depression.

## Method

### Trial design and assignment

This study is designed as a single-center, assessor-blind, randomized control trial. The subjects are categorized based on age (15–24 years old, 25–34 years old) and gender (male, female). Subsequently, the subjects are randomly allocated to three groups, namely the control group, the exercise group, and the exergames group, in a 1:1 allocation ratio. To ensure equal distribution among the three groups, a randomized block design is employed utilizing permuted blocks with randomized block lengths of 6. Group assignments are determined through the use of computer-generated random numbers, with assignment codes concealed within sequentially numbered, sealed, opaque envelopes. These envelopes are securely stored in a locked location within the study research office and are only opened upon a subject meeting the inclusion criteria.

### Objectives

The study's principal objective is to assess the cognitive advantages of a new intervention, specifically exergames, in adolescents diagnosed with depression. The secondary objective is to compare the effectiveness of exergames and exercise on cognition in patients. Lastly, the study aims to compare the clinical outcomes of exergames and exercise on mood and sleep enhancement in patients with depression.

### Blinding

Because participants are aware of whether they exercise, it is not possible to blind them to their treatment allocation. However, none of the researchers who collect and statistically analyze the data are aware of the patient assignments. Strict instruction is given to the patients not to reveal their group allocation to the test personnel.

### Ethics

**Fig. 1:**
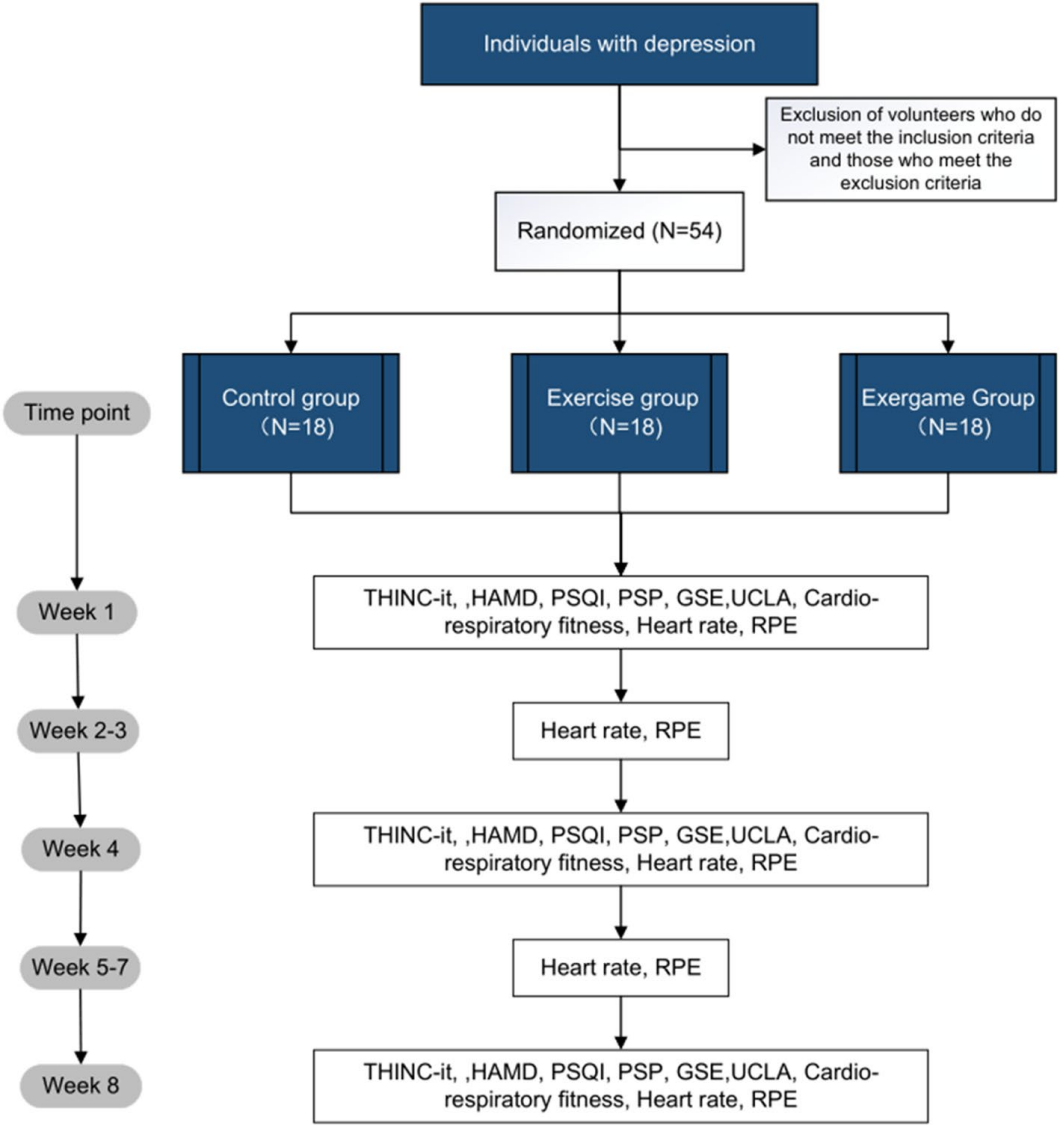
Trial flow diagram. PSQI, Pittsburgh Sleep Quality Index; PSP, Personal and Social Performance Scale; HAMD, Hamilton Depression Scale; GSES, General Self-Efficacy Scale – Schwarzer; UCLA, The University of California, Los Angeles Loneliness Scale; RPE, The Borg Rating of Perceived Exertion scale

The present investigation adheres to the principles outlined in the Declaration of Helsinki and has been authorized by the Ethics Committee of the West China Hospital of Sichuan University. In the event that modifications to the protocol are required, the investigators promptly inform the relevant ethics committees or institutional review boards. Currently, the protocol version is 1.0, which is used for the initial protocol. In this study, written informed consent forms are obtained from all participants, while participants under the age of 16 require the additional signature of their guardians. Participants are informed of the voluntary nature of their involvement and their right to withdraw from the experiment at any time. To protect their privacy, personal information is anonymized and securely stored in locked filing cabinets. All subject information is treated confidentially and solely utilized for the purposes of the study. The study’s investigators have no funding, financial relationships, or conflicts of interest to disclose  (Fig. 1).

### Participants

This study recruits 54 patients diagnosed with depression (in the acute or postacute phase) from the Mental Health Center of West China Hospital of Sichuan University. The remaining 54 depressed patients are randomly assigned to one of three groups. All individuals are interviewed using the Structured Clinical Interview for Diagnostic and Statistical Manual of Mental Disorders, fifth edition (DSM-V) clinical edition (SCID-5-CV). Diagnosis is made by a consensus of two experienced clinicians from the data obtained in this interview. Several criteria are considered when selecting participants. The inclusion/exclusion criteria are outlined in Table 1. The Inclusion Criteria are as follows: (1) Age: Participants must be between the ages of 15 to 34 years; (2) Nationality: Participants are of Han ethnicity; (3) Handedness: Participants are right-handed; (4) Diagnosis: Participants are the criteria for a DSM-5 diagnosis of moderate Depression according to the Structured Clinical Interview for DSM-5 (SCID-5-CV); (5) Drug Use: Participants are using antidepressants; (6) Informed Consent: Participants sign a written informed consent form, and guardians of participants aged under 16 years must also sign a written informed consent form. The exclusion criteria are as follows: (1) Suffering from colour blindness, colour weakness, deafness, stuttering, and other conditions; (2) Pregnant and parturient within a year; (3) With diseases such as neurological diseases, and organic diseases of the brain; (4) With diseases such as hypertension and heart disease and other conditions that are associated with a higher risk of exercise; (5)Cardiorespiratory Fitness: the results of the 3-Minute Step Test are poor; (6) With a previous suicide attempt or plan of committing suicide.

**Table 1:** Inclusion and exclusion criteria of patients with depression

Inclusion Criteria	Exclusion criteria
Age:15–34 years	Suffering from colour blindness, colour weakness, deafness, stuttering, and other conditions
Nationality: Han	Pregnant and parturient within a year
Handedness: right-handedness	With diseases such as neurological diseases, and organic diseases of the brain
Diagnosis: Meets criteria for a DSM-5 diagnosis of moderate Depression according to SCID-5-CV	With diseases such as hypertension and heart disease and other conditions that are associated with a higher risk of exercise
Drug Use: use antidepressants	Cardiorespiratory Fitness: the results of the 3-Minute Step Test are poor
Informed Consent: sign a written informed consent form, and guardians of participants aged < 16 years also signed a written informed consent form	With a previous suicide attempt or plan of committing suicide

### Procedure

**Fig. 2:**
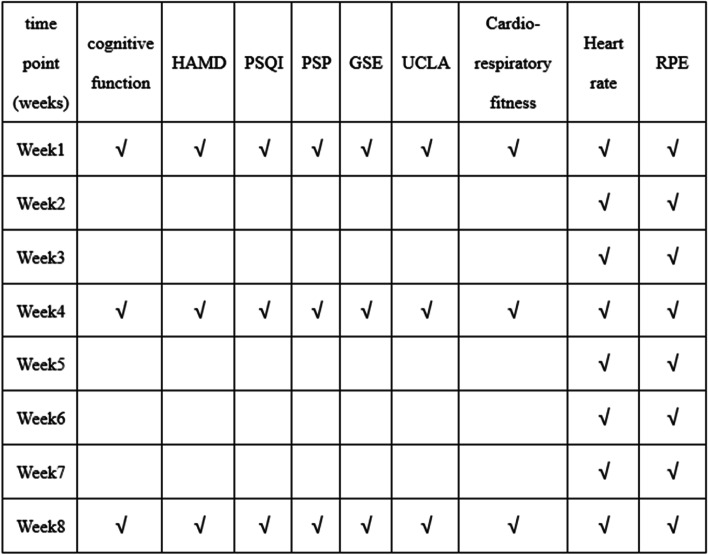
Assessment items and assessment time. PSQI, Pittsburgh Sleep Quality Index; PSP, Personal and Social Performance Scale; HAMD, Hamilton Depression Scale; GSES, General Self-Efficacy Scale – Schwarzer; UCLA, The University of California, Los Angeles Loneliness Scale; RPE, The Borg Rating of Perceived Exertion scale

Volunteers suitable for this study are first screened by inclusion and exclusion criteria, which included performing the cardiorespiratory fitness test. Suitable volunteers sign an informed consent form and are randomly assigned to each group. First, participants are assessed at baseline. Second, patients entering the Exergames and Exercise groups are instructed to come to the hospital twice a week for the first two weeks and three times each week for the last six weeks. Prior to arrival, participants are requested to observe a minimum fasting period of 1.5 h and don comfortable, loose-fitting attire and sneakers. Additionally, volunteers undergo assessments for cognitive function, Hamilton Depression Scale (HAMD), Pittsburgh Sleep Quality Index (PSQI), Personal and Social Performance Scale (PSP), General Self-Efficacy Scale (GSE), and The University of California, Los Angeles Loneliness Scale (UCLA) on the day before they participate in treatment, at the end of week 4 and the end of week 8. Heart rate is monitored during each exercise session using a wearable device, and RPE is assessed at the end of each exercise session. The assessment items and the assessment times are shown in Fig. 2. The primary outcome is cognitive function. The secondary outcomes are mood symptoms and sleep quality. The other outcomes are social functioning, self-efficacy, subjective feelings of loneliness and social isolation, the objective exercise intensity, and subjective fatigue and intensity of exercise.

### Interventions

Patients with depression who enter the control group are treated according to depression treatment guidelines. Treatment methods include only medications. Patients with depression who enter the exergames group receive 8 weeks of exergames program intervention (Table 2) and drug treatment. The exergames are Ring Fit Adventure [18] and Just Dance [19]. Exergames are multicomponent exercises, and the main type of exercise is aerobic exercise. The first phase of the exergames programs is the adaptation phase, which lasts for 2 weeks. The frequency of exergames is 2 times a week, the intensity of exercise is low, and the duration of each exercise is 30–50 min. The second phase of the exergames programs is the hardening stage, which lasts for 6 weeks. The frequency is 3 times a week, the intensity is moderate, and the duration is 50–70 min. Patients with depression who enter the exercise group receive 8 weeks of traditional exercise program intervention (Table 3) and drug treatment. The traditional exercise program is running and cycling, and the type of exercise is aerobic. The first phase of the traditional exercise programs is the adaptation phase, which lasted for 2 weeks. The frequency is 2 times a week, the intensity is low, and the duration of each exercise is 30–50 min. The second phase of the traditional exercise program is the intensive phase, which lasts for 6 weeks. The frequency is 3 times a week, the intensity is moderate, and the duration of each exercise is 50–70 min.

**Table 2:** The 8-week exergame program

Exergames program	
The total duration of treatment: 8 weeks	
The first phase of the exergame programs	
Adaptation stage	Weeks 1–2
exergames	Ring Fit Adventure and Just Dance
Exercise frequency	2 times per week
Exercise intensity	low intensity
Exercise time	30 min-50 min each time
Exercise type	multicomponent exercise (the main type of exercise is aerobic)
The second phase of the exergame programs	
Hardening stage	Week 3–8
exergames	Ring Fit Adventure and Just Dance
Exercise frequency	3 times per week
Exercise intensity	moderate intensity
Exercise time	50 min-70 min each time
Exercise type	multicomponent exercise (the main type of exercise is aerobic)

**Table 3:** The 8-week traditional exercise program

Traditional exercise program	
The total duration of treatment: 8 weeks	
The first phase of the traditional exercise programs	
Adaptation stage	Weeks 1–2
exercise	Running and cycling
Exercise frequency	2 times per week
Exercise intensity	low intensity
Exercise time	30 min-50 min each time
Exercise type	aerobic exercise
The second phase of the traditional exercise programs	
Hardening stage	Week 3–8
exercise	Running and cycling
Exercise frequency	3 times per week
Exercise intensity	moderate intensity
Exercise time	50 min-70 min each time
Exercise type	aerobic exercise

### Sample size calculation

The sample size (n) is determined by G*Power software version 3.1.9.2 (Universität Düsseldorf, Germany). The level of significance is set at α = 0.05, and the statistical tests are two-tailed. For statistical power, 1- β is set to 0.8. There are three groups in this study, and each sample is repeatedly measured 3 times. The total sample size is 45 using between factors, two-factor repeated measurement analysis of variance (ANOVA). Assuming a dropout rate of 25%, a final total sample size of 54 is calculated (18 per group).

### Data collection

In this project, the testing is conducted by a trio of assessors who undergo a rigorous week-long training in professional evaluation. The training encompasses comprehensive instructions and demonstrations of all testing procedures and ratings, and is delivered through a combination of face-to-face instruction and practical operation guidance. The assessors' agreement is assessed using the kappa coefficient (κ) statistical test, with a kappa value exceeding 0.8 deemed satisfactory.

#### Thinc-Integrated Tool (THINC-it)

Cognitive function is tested using the Thinc-Integrated Tool (THINC-it). It is a third-party mobile app that comprises 5 quick interactive tests to assess cognitive function, including executive function, learning and memory, processing speed, and attention. THINC-it makes use of the One-Back Test, the Choice Reaction Time task, the Trail Making Test Part B, the Digit Symbol Substitution Test, and the Perceived Deficits Questionnaire for Depression 5-item version (Table 4). The table shows the specific assessment time and the corresponding assessment items.

**Table 4:** The tests in THINC-it

The tests in THINC-it	Ability
PDQ-5	cognitive function (Self-evaluation)
CRT	processing speed
N-back	working memory
DSST	executive function sustained attention and processing speed
TMT-B	attention, executive function (set shifting), and cognitive flexibility

*PDQ-5* The Perceived Deficits Questionnaire for Depression 5-item version, *CRT* The Choice Reaction Time task, *N-back* The One-Back Test, *DSST* The Digit Symbol Substitution Test, *TMT-B* The Trail Making Test Part B

#### Hamilton Depression Scale (HAMD)

The Hamilton Depression Scale (HAMD) assesses mood symptoms. It better reflects the severity of the disease in depression. The HAMD scale is widely used in the clinical assessment of depression. The scale is compiled by Hamilton in 1960, and the Chinese version has good reliability and validity [20]. In this study, the HAMD-24 with 7 dimensions and 24 items is used. The scale has a scoring system of five levels from 0 to 4 for most items and a range of two levels from 0 to 2 for a few items.

#### Pittsburgh Sleep Quality Index (PSQI)

The Pittsburgh Sleep Quality Index Scale is used to measure sleep quality over the past 30 days, and the Chinese version has been validated to have good reliability and validity [21]. It is a self-report with 7 sections including subjective sleep quality, sleep onset latency, total sleep duration, sleep efficiency, sleep disturbances, sleep medication use, and daytime dysfunction. The total score ranges from 0–21, with a total score greater than 16 indicating poor sleep quality.

#### Personal and Social Performance Scale (PSP)

The Personal and Social Performance Scale is a clinician's tool for assessing a patient's social functioning in four main areas: self-care, social activities, social relationships, and disruptive and aggressive behaviours. The test score can range from 1 to 100, with higher scores indicating better functioning.

#### General Self-Efficacy Scale (GSE)

The General Self-Efficacy Scale is developed by Schwarzer and Jerusalem (1995) to measure self-efficacy. The scale contains 10 items with a total score range of 10 to 40; the higher the score is, the greater the self-efficacy. The Chinese version is originally developed by Zhang and has good reliability and validity [22].

#### The University of California, Los Angeles Loneliness Scale (UCLA)

The University of California, Los Angeles Loneliness Scale (version 3) [23] consists of 20 items to assess subjective feelings of loneliness and social isolation. Participants rate how often they felt the way described in the items using a four-point Likert scale ranging from "never" to "often". The higher the total score, the greater the loneliness.

#### Wearable devices

During the test, participants are equipped with a single wearable physical activity monitor on their left wrist. HUAWEI WATCH GT2 is used to monitor the heart rate of participants during exercise and to assess the objective exercise intensity of participants.

#### The Borg Rating of Perceived Exertion scale (RPE)

The Borg Rating of Perceived Exertion scale is used to assess participants' subjective fatigue and intensity of exercise. The scale ranges from 6 to 20, with higher scores indicating more fatigue and greater exercise intensity.

#### Cardiorespiratory fitness test: 3-min step test

In order to ensure the safety of the participants and minimize potential risks, a 3-min step test is administered to assess their cardiorespiratory endurance. Individuals with inadequate cardiopulmonary function are deemed ineligible for inclusion in this study.

- The height of the male step is 40 cm, and the height of the female step is 35 cm. According to the different heights of men and women, the step can also make appropriate adjustments. The test can be carried out in the following steps. Participants maintain an appropriate rhythm of stepping and jumping during the test. They walk up and down the steps 30 times according to the metronome's rhythm of 120 beats per minute. The upper body and legs must be straight after each step up and down, without bending their knees.
- Participants sit down immediately after the test and measure their pulse during three recovery periods after the exercise: 1 min to 1 min 30 s, 2 min to 2 min 30 s, and 3 min to 3 min 30 s.

Scoring is based on guidelines of the age-adjusted standards (Supplementary Table 1) published by the Young Men's Christian Association (YMCA). The higher the score, the better the cardiopulmonary function.

### Data management

The clinical record form (CRF) data, which is completed by one assessor, undergoes a thorough examination by another assessor to ensure accuracy across a spectrum of data values. To mitigate the possibility of data entry errors, a double data entry system is employed. All data is securely stored in a computer database, with exclusive access granted to the research leader for all data and the data analyst for solely deidentified data. The trial results are disseminated to both participants and the public through publications and conference presentations. No ancillary or posttrial care is planned.

### Data monitoring

The research leader is responsible for ensuring that the data entered are accurate, complete, and timely. The study's data monitoring committee consists of three independent medical doctors with no conflicts of interest. Monitoring staff ensures that the data documented in both the CRF and the source document are consistent. No regular auditing is planned. The expected risk in this research is low. A response plan for all anticipated possible adverse events is being developed, adverse events are being recorded, and the study leader and ethics committee will be informed.

### Statistical analysis

The per-protocol analysis (PP) principle is strictly followed in all of our analyses. A chi-square (χ 2) test is employed to assess the frequency of differences in qualitative characteristics (e.g., gender) among the three groups. The Shapiro‒Wilk test is utilized to examine normality. In the event of a normal distribution, the two-factor repeated measures analysis of variance (ANOVA) test is applied. However, if the distribution deviates from normality, the Scheirer-Ray-Hare test is utilized.

## Discussion

Recent research indicates that exercise, whether utilized as a supplementary treatment or independently, has the potential to enhance the quality of sleep [24], alleviate symptoms of depression, and improve cognitive performance [25] in patients with depression. Further research is necessary to establish the impact of exergames on enhancing cognitive function in individuals with depression, given that it is a novel mode of physical activity. Additionally, it is imperative to investigate whether exergames yield superior outcomes in cognitive function compared to conventional exercise among adolescents with depression. The present study is centered on the assessment of the cognitive advantages of a new intervention, namely exergames, in adolescents afflicted with depression. The study aims to determine whether the cognitive benefits of exergames surpass those of exercise in enhancing cognitive function in teenagers with depression. Furthermore, this study endeavors to compare the efficacy of both adjunctive treatments in enhancing mood and sleep quality in individuals with depression. 

### Strengths and limitations

There are several strengths to this work. Specifically, it conducts a preliminary investigation into the potential of a novel cognitive intervention, namely exergames, for the treatment of depression. Prior research on the cognitive benefits of exergames has primarily focused on the elderly population. However, this study endeavors to examine the impact of exergames on cognitive function in adolescents with depression. Moreover, this study compares for the first time the efficacy of exergames and traditional exercise on the cognition of adolescents with depression. The research encompasses a thorough evaluation of various cognitive domains, such as executive function, learning and memory, processing speed, and attention. This study is subject to several limitations. Firstly, the small sample size presents a clear constraint on the study's validity. To further validate the results, a larger sample size will be required in future research. Secondly, the study lacks follow-up on cognitive functions to ascertain the duration of the effects of exergames and exercise interventions on cognitive functions. Thirdly, the study lacks an in-depth mechanistic discussion, and future research could incorporate genetic testing, intestinal flora, EEG, MRI, and other analytical methods to investigate the causes of cognitive function enhancement through exergames intervention.

## Supplementary Information

**Additional file 1: Supplementary Table 1.** Table of 3-Minute Step Test index values and cardiopulmonary function.

## Data Availability

Not applicable.

## Abbreviations

HAMD: Hamilton Depression Scale
PSQI: Pittsburgh Sleep Quality Index
PSP: Personal and Social Performance Scale
GSE: General Self-Efficacy Scale
UCLA: The University of California, Los Angeles Loneliness Scale
RPE: The Borg Rating of Perceived Exertion scale
FDA: Food and Drug Administration
DSM-V: Diagnostic and Statistical Manual of Mental Disorders, fifth edition
SCID-5-CV: Structured Clinical Interview for DSM-5, clinical edition
ANCOVA: Analysis of variance
THINC-it: Thinc-Integrated Tool
PDQ-5: The Perceived Deficits Questionnaire for Depression 5-item version
CRT: The Choice Reaction Time task
N-back: The One-Back Test
DSST: The Digit Symbol Substitution Test
TMT-B: The Trail Making Test Part B
YMCA: The Young Men's Christian Association
