# Supplementary material for: Exergames improves cognitive functions in adolescents with depression: study protocol of a prospective, assessor-blind, randomized controlled trial
Source: BMC Psychiatry. 2023 Jul 13;23:507. doi: 10.1186/s12888-023-04967-7 (PMC10339627; doi:10.1186/s12888-023-04967-7)
Supplement: Supplementary file 1 — Additional file 1: Supplementary Table 1. Table of 3-Minute Step Test index values and cardiopulmonary function. [file 12888_2023_4967_MOESM1_ESM.docx]

Supplementary Table 1. Table of 3-Minute Step Test index values and cardiopulmonary function.

| Age  Standard | 18-25 | | 26-30 | | 31-40 | | 41-50 | | 51-60 | |
| --- | --- | --- | --- | --- | --- | --- | --- | --- | --- | --- |
|  | Male | female | Male | female | Male | female | Male | female | Male | female |
| Excellent | >79 | >78 | >78 | >78 | >78 | >78 | >79 | >79 | >79 | >78 |
| Good | 71-79 | 70-78 | 70-78 | 70-78 | 69-78 | 69-78 | 69-79 | 69-79 | 69-79 | 69-79 |
| Average | 50-70 | 50-69 | 50-69 | 49-69 | 50-68 | 49-68 | 50-68 | 47-67 | 48-68 | 46-66 |
| Below Average | 41-49 | 39-49 | 40-49 | 39-48 | 39-49 | 38-48 | 38-49 | 36-46 | 36-47 | 34-45 |
| Poor | <41 | <39 | <40 | <39 | <39 | <38 | <38 | <36 | <36 | <34 |
